# Supplementary material for: Neurocognitive Correlates of Clinical Decision Making: A Pilot Study Using Electroencephalography
Source: Brain Sci. 2023 Nov 30;13(12):1661. doi: 10.3390/brainsci13121661 (PMC10741622; doi:10.3390/brainsci13121661)
Supplement: Supplementary file 1 [file brainsci-13-01661-s001.zip › brainsci-2711915-supplementary.pdf]

Supplementary materials

Mock applied board-style clinical case stem, questions, and the assessment form.

Stem Question

A 22-year old female injured her right leg this morning while ice-skating and presents for repair of an open tibial fracture. She has a long history of asthma, takes Singulair, Albuterol and Flovent daily and periodically takes oral steroids. She had an upper respiratory infection and exacerbation of baseline wheezing one week ago and took oral steroids for 3-4 days that she is no longer taking. She says she frequently wheezes; today after her injury she says she is more short-of-breath than at baseline. Blood pressure is 142/78 mm Hg, pulse is 112 bpm, respirations are 32, and temperature is 36.8 degrees centigrade. She has diffuse wheezing with good air entry currently and is complaining of 10/10 pain in her right leg and open wound is evident. The patient drank a smoothie at 9AM. It is currently 1PM.

Fellow or Resident Name/Study Number ______________________/___

Section A: Core Question – 12 minutes

Topic & questions			
**Evaluation of respiratory status**	**Y**	**M**	**N**
What further evaluation of respiratory status is indicated?<br><br>Would you suggest further medical therapy? What & why?<br><br>What is the impact of URI on anesthetic risk? Impact of wheezing on anesthetic risk?			
**Choice of anesthetic**	**Y**	**M**	**N**
What anesthetic technique would you use for this case? Explain. Would regional anesthesia be a reasonable choice? Why? Why not? What agents would you use for induction? Why?<br><br>How does the recent URI affect your decision? How does her asthma affect your decision?<br><br>She is afraid of being awake for the procedure. What is your response? Consideration of NPO status?			
**Pain Control**			
What are your plans for pain control? Multimodal? Would regional be a reasonable choice? Which? Why? Patient requests epidural. Would a spinal be appropriate? What about peripheral nerve block? Which? What local anesthetic agent would you use for a subarachnoid block? Explain your choice. Is epinephrine necessary? Explain What level of block is needed for this procedure?<br><br>After completion of epidural dosing, she begins to complain of numbness in her fingers. DDx? Rx?			
**Bronchospasm under general anesthesia**			
Anesthesia is maintained with Sevoflurane and nitrous/oxygen by ETT. Fifteen minutes into the case, the ETCO2 increases. DDx? Marked wheezing and heart rate increases to 122 bpm. What is your evaluation and management?<br><br>While attempting to manage, her end-tidal CO2 falls to 22 mm Hg. DDx? Rx? Albuterol? Epinephrine? Deepen anesthetic?<br><br>Should you cancel the case?			
**Intraoperative Steroid Dosing**			
Should this patient be given stress doses of steroids? Why? What evidence? How much will you give? What dosing regimen?<br><br>Should this patient be given steroids for other purposes? For what? Why?			

Section B: Extra Question – 3 minutes

**Topic & questions**			
**Myasthenia Gravis**	**Y**	**M**	**N**
A 45 yo woman with myasthenia gravis is scheduled for an open cholecystectomy due to acute cholecystitis. She has had MG for 5 years treated with pyridostigmine bromide, total dose 720 mg/day. She has not had thymectomy and has mild bulbar symptoms.<br><br>What would be your anesthetic of choice of this patient? Why? Regional or GA?<br><br>Would you use muscle relaxant? If so which? Sux v non-depolarizer? What is your plan for reversal? Case is scheduled at 2PM, does this impact your choice?<br><br>One hour after Cis-atracurium, TOF = 0; how to proceed?<br><br>In PACU the patient is weak, how do you distinguish between myasthenic and cholinergic crisis?			

Quantitative Score

#Yes ________

#Maybe ________

#No __________

Qualitative Score (check one or more)

No deficient attributes

Deficient judgment

Deficient adaptability

Deficient application of knowledge

Deficient organization/presentation
